# Hippocampal and Cerebral Blood Flow after Exercise Cessation in Master Athletes

**DOI:** 10.3389/fnagi.2016.00184

**Published:** 2016-08-05

**Authors:** Alfonso J. Alfini, Lauren R. Weiss, Brooks P. Leitner, Theresa J. Smith, James M. Hagberg, J. Carson Smith

**Affiliations:** Department of Kinesiology, University of MarylandCollege Park, MD, USA

**Keywords:** aerobic fitness, arterial spin labeling, athlete, cerebral blood flow, cerebrovascular health, healthy older adults, hippocampus, MRI

## Abstract

While endurance exercise training improves cerebrovascular health and has neurotrophic effects within the hippocampus, the effects of stopping this exercise on the brain remain unclear. Our aim was to measure the effects of 10 days of detraining on resting cerebral blood flow (rCBF) in gray matter and the hippocampus in healthy and physically fit older adults. We hypothesized that rCBF would decrease in the hippocampus after a 10-day cessation of exercise training. Twelve master athletes, defined as older adults (age ≥ 50 years) with long-term endurance training histories (≥15 years), were recruited from local running clubs. After screening, eligible participants were asked to cease all training and vigorous physical activity for 10 consecutive days. Before and immediately after the exercise cessation period, rCBF was measured with perfusion-weighted MRI. A voxel-wise analysis was used in gray matter, and the hippocampus was selected *a priori* as a structurally defined region of interest (ROI), to detect rCBF changes over time. Resting CBF significantly decreased in eight gray matter brain regions. These regions included: (L) inferior temporal gyrus, fusiform gyrus, inferior parietal lobule, (R) cerebellar tonsil, lingual gyrus, precuneus, and bilateral cerebellum (FWE *p* < 0.05). Additionally, rCBF within the left and right hippocampus significantly decreased after 10 days of no exercise training. These findings suggest that the cerebrovascular system, including the regulation of resting hippocampal blood flow, is responsive to short-term decreases in exercise training among master athletes. Cessation of exercise training among physically fit individuals may provide a novel method to assess the effects of acute exercise and exercise training on brain function in older adults.

## Introduction

Endurance exercise training (exercise) produces physiological adaptations that enhance aerobic fitness and cardiovascular health (Brooks et al., 1996). Consistent exercise effectively augments the maximal rate of oxygen consumption ($\overset{\cdot }{\text{V}}$O_2max_) centrally, by increasing cardiac output, and/or peripherally by widening the arterial-venous oxygen (A-$\overset{\cdot }{\text{V}}$O_2_) difference (Seals et al., 1981). $\overset{\cdot }{\text{V}}$O_2max_ is the gold-standard index of cardiorespiratory fitness and is highly correlated with both morbidity and mortality (Hoekstra et al., 2008; Sawada et al., 2012), with greater fitness status associated with a reduced risk of chronic disease and a longer lifespan. In addition to enhancing the function of the cardiovascular system, exercise has been shown to increase bone density, improve muscle quality, and protect against metabolic dysfunction (Brooks et al., 1996). Conversely, when the exercise stimulus is removed many of these systemic adaptations rapidly dissipate (Mujika and Padilla, 2000a, 2001a,b), thereby increasing the potential for adverse health effects. For example, 20 days of bed rest immobilization resulted in a substantial 28% decrease in $\overset{\cdot }{\text{V}}$O_2max_ (Saltin et al., 1968); a prolonged detraining period reduced muscle fiber capillarization and oxidative enzyme activity (Klausen et al., 1981); and a 10-day period of physical inactivity was related to the development of impaired glucose tolerance and insulin resistance (Rogers et al., 1990).

A growing body of empirical evidence supports the notion that exercise also robustly affects the human brain. Multimodal neuroimaging studies, including both structural and functional MRI, have helped elucidate the brain's complex neurobiological response to exercise. These exercise-induced effects include cytoarchitectonic modifications (Erickson et al., 2009; Smith et al., 2014; ten Brinke et al., 2015); altered patterns of neural activity (Smith et al., 2013); and improved performance across the cognitive domains (Tomporowski, 2003; Kramer et al., 2005; Davranche and McMorris, 2009; Chapman et al., 2013). The hippocampus, a subcortical brain structure well known for its role in learning and memory, has shown neurotrophic effects as the result of exercise training in humans and animal models (van Praag et al., 1999; Pereira et al., 2007; Erickson et al., 2009). Exercise interventions in humans have been shown to affect hippocampal-dependent cognition and to increase hippocampal blood perfusion (Pereira et al., 2007) and volume (Erickson et al., 2009). While the effects of detraining have been reported in peripheral physiological systems, the effects of detraining on brain function, and on cortical and hippocampal blood flow, have not been reported.

A key unanswered question, and the primary aim of this study, was to determine how short-term exercise cessation impacts cerebrovascular function in healthy highly physically active and physically fit older adults. To accomplish this goal we measured the resting cerebral blood flow (rCBF) of master athletes both before and immediately after 10 days of exercise cessation. To quantify rCBF we employed pseudo-continuous arterial spin labeling (pCASL), a perfusion-weighted MRI technique. Our hypotheses were twofold. We predicted (1) that 10 days of physical inactivity would alter rCBF in areas known to be susceptible to age-related decline (Greicius et al., 2004; Buckner et al., 2005), and (2) that detraining would decrease hippocampal blood flow, which we chose as an *a priori* region of interest (ROI).

## Methods

### Participants

Our unique study sample consisted entirely of master athletes, which were defined as a sub-group of highly trained healthy older adults who regularly engaged in endurance exercise. Master athletes ranged in age between 50 and 80, and were recruited from local Washington D.C. area running clubs. These individuals had an endurance exercise history of at least 15 years and had recently competed in regional and national endurance events. Personalized training regimens must have entailed at least 4 h of high intensity endurance training per week.

This study was approved by the Institutional Review Board, and written informed consent was obtained from all participants, in accordance with the Helsinki declaration. A telephone screen was used to determine eligibility. Those who met inclusion/exclusion criteria (see below) and agreed to participate completed the following: anthropometric measurement, a maximal intensity treadmill/electrocardiography test, DEXA (Dual-energy X-ray absorptiometry) body composition assessment, neuropsychological testing, and fMRI scanning (Table 1 shows descriptive data for variables examined at baseline only).

**Table 1:** **Descriptive participant data collected at baseline**.

	**Mean (SD)**	**Maximum**	**Minimum**
Age (y)	61 (7.8)	71	51
$\overset{\cdot }{\text{V}}$O_2max_ (ml/kg/min)	46.2 (5.50)	54.0	36.1
Training history (yrs)	29 (6.3)	36	20
Body fat (%)	25.2 (3.6)	29.9	18.55
Fat mass (kg)	17.8 (4.0)	23.7	11.99
Lean mass (kg)	50.3 (9.0)	63.44	34.5
BMI (kg/m^2^)	23.4 (3.5)	29.4	19.5
SBP (mm/Hg)	117.6 (16.5)	146	104
DBP (mm/Hg)	70 (8.90)	82	60
MAP (mm/Hg)	85.9 (10.4)	103.3	74.7
MMSE	28.7 (1.1)	30	27

*$\overset{\cdot }{V}$O_2max_, maximal oxygen consumption; BMI, body mass index; SBP, systolic blood pressure; DBP, diastolic blood pressure; MAP, mean arterial pressure; MMSE, Mini Mental State Exam*.

### Inclusion and exclusion criteria

Individuals were excluded if they had a BMI ≥ 30, reported being a smoker (within the past 5 years), or had a history of heart attack, stroke, lung disease, chronic obstructive pulmonary disease, peripheral vascular disease, heart disease, liver disease, kidney disease, anemia, or diabetes. Individuals taking prescription medication for hypertension were also deemed ineligible. Women participants must have been post-menopausal for at least 2 years and must not have used hormone therapy during the previous year. Additionally, individuals were excluded if they presented any absolute contraindications to MRI. During the initial telephone screening 21 persons were deemed ineligible to participate in this study. Of the 12 individuals who qualified for participation, nine (7 men, ~89% Caucasian) were included in the final analysis. One individual was removed due to irregular ECG activity during the graded treadmill test; another because of dental work that severely distorted the MR signal; and a third for failure to achieve $\overset{\cdot }{\text{V}}$O_2max_ during the graded treadmill exercise test.

### Neuropsychological testing

Prior to the baseline MRI scan, all participants were administered the Mini Mental State Exam (MMSE) (Folstein et al., 1975), which is a 30-point questionnaire used to screen for global cognitive impairment and dementia. Additionally, after both the baseline and follow-up MRI scan all participants were administered the semantic verbal fluency test (Monsch et al., 1992). This test required participants to list as many words as possible from a given category in 60-s (fruits and animals, order counterbalanced).

### $\overset{\cdot }{\text{v}}$O_2max_ testing

Cardiorespiratory fitness was determined by assessing $\overset{\cdot }{\text{V}}$O_2max_ during a graded treadmill test with indirect calorimetry (Quark, Cosmed USA). Exercise tests were conducted utilizing a protocol we have used numerous times previously in older athletes, and included standard ECG monitoring (Rogers et al., 1990; Brooks et al., 1996). The exercise test continued until maximal effort or exhaustion was achieved. For exercise tests to be considered maximal, participants had to reach both a plateau in $\overset{\cdot }{\text{V}}$O_2max_ with increasing workload and a respiratory exchange ratio >1.1. The highest $\overset{\cdot }{\text{V}}$O_2_ attained during the test was recorded as $\overset{\cdot }{\text{V}}$O_2max_. The maximal exercise test was conducted several hours after the baseline MRI scan.

### Body composition assessment

Body composition was measured using dual energy x-ray absorptiometry (DEXA) (DXA; Prodigy, LUNAR Radiation Corp).

### Exercise cessation period

MRI scanning occurred at two time points, baseline and immediately after the 10-day exercise cessation. Participants were asked to refrain from exercise during the 12 h preceding the baseline assessment. The exercise cessation commenced 72 h after the baseline scan, at which time all exercise training was stopped. During this period participants were asked to refrain from exercise and all other forms of vigorous physical activity. The abstention from exercise during the 10-day period was verified frequently by telephone conversations with the participants and during their final testing. The second MRI scanning session occurred on the morning after the last day of the exercise cessation period, and before participants resumed their training regimens.

### MRI acquisition

All MRI data were acquired with a Siemens 3.0 Tesla MR system (Magnetom Trio Tim Syngo, Munich, Germany). A 32-channel head coil was used for radio frequency (RF) transmission and reception. Foam padding was positioned within the head coil to minimize patient motion. A high-resolution T1-weighted anatomical image was acquired for co-registration with the following sequence parameters: Magnetization Prepared Rapid Acquisition of Gradient Echo (MPRAGE), matrix = 256, field-of-view (FOV) = 230 mm, voxel size = 0.9 × 0.9 × 0.9 mm, slices = 192 (sagittal plane, acquired right to left), slice thickness = 0.9 mm, repetition time (TR) = 1900 ms, echo time (TE) = 2.32 ms, inversion time (TI) = 900 ms, flip angle = 9°, sequence duration = 4:26 min. The pCASL data were acquired using the following sequence parameters: single-shot gradient echo planar images, matrix = 64, FOV = 210 mm, voxel size = 3.28 × 3.28 × 6.0 mm, slices = 20 (axial plane, acquired in ascending order), slice thickness = 5.0 mm, gap between slices = 1 mm, single slice acquisition time = 48 ms, label duration = 1500 ms, post-label delay = 1000–1912 ms, TR/TE = 4000/19 ms, volumes = 140, number of label/control pairs = 70, flip angle = 90°, RF blocks = 80, RF pulses = 20, gap between pulses = 360 μs, bandwidth = 3004 Hz/Px, and sequence duration = 9:28 min. Additionally, a concatenated series of control volumes stacked in the time dimension was used as the proton density (PD) image for perfusion calibration.

### MRI data preprocessing

The pCASL and PD images were realigned to the first volume of the image time series for motion correction (Cox, 1996). Using pairwise subtraction, a perfusion-weighted image was derived from the motion-corrected interleaved (control—tag) volumes, and this image was corrected for slice-timing delay (Woolrich et al., 2009).

Using FSL's BASIL (Bayesian Inference for Arterial Spin Labeling, FMRIB Software Library v5.0, Oxford, UK) (Woolrich et al., 2009), a reference mask from the T1-weighted anatomical image was used to isolate and sample the cerebral spinal fluid (CSF) within the ventricles of the PD image. The CSF sample was used to compute the magnetization equilibrium (M0) of tissue, which was further used to obtain the magnetization equilibrium of arterial blood (M0a) (Woolrich et al., 2009).

Buxton's general model for kinetic inversion was used to estimate absolute CBF (ml/100g/min) (Buxton et al., 1998), and included both Bayesian inferences and the following parameters (Chappell et al., 2009): ΔM (proportional magnetization change represented by the perfusion-weighted image), T1_blood_ (longitudinal relaxation time of blood = 1650 ms), T1_tissue_ (longitudinal relaxation time of tissue = 1300 ms), α (labeling efficiency = 0.85), M0_a_ (magnetization equilibrium of arterial blood), BAT (bolus arrival time = 1300 ms), Bolus (label duration = 1500 ms), PLD (post label delay = 1000 ms), and λ (blood/brain partition coefficient of GM = 0.98 ml/g) (Buxton et al., 1998; Alsop et al., 2014). The rendered CBF map was spatially smoothed using an adaptive technique, which combined neighboring voxel signals on an intensity-dependent basis, while preserving the non-linear kinetics where smoothing was unnecessary (Groves et al., 2009; Chappell, 2014). Due to the inherently low spatial resolution of the perfusion image, partial volume error correction was performed to improve the accuracy of CBF estimation (Chappell et al., 2011; Chappell, 2014). This process effectively calculated separate gray matter and white matter perfusion using tissue specific partial volume estimates in each voxel (Chappell et al., 2011; Chappell, 2014). Co-registration and normalization was performed with SPM8 (Hall and Degenhardt, 2008; SPM, University College, London, UK), using the PD image as the reference to which the CBF map was aligned. The output parameters were used to transform the CBF map and gray matter estimates to standard space, which effectively up-sampled the images to a 2 mm^3^ isotropic voxel resolution. Normalized gray matter estimates were merged together and used as a mask for the voxel-wise analysis.

### Gray matter voxel-wise analysis

A voxel-wise analysis, restricted to voxels within the gray matter mask, was conducted to explore the effect of exercise cessation on rCBF. Statistical parametric maps were produced (Cox, 1996), indicating where rCBF had significantly changed over time. We used AFNI's 3dClustsim program (on the 2 mm^3^ data) to control for the effects of multiple comparisons and reduce the likelihood of a Type-I error. This analysis tool used Monte Carlo simulations to establish a family-wise error (FWE) corrected probability threshold at both the voxel (*p* < 0.05) and cluster (α < 0.05) level (Cox, 1996). Using first-order nearest neighbor clustering, we maintained results at a minimum cluster size of ≥480 mm^3^. To further illustrate the results of the voxel-wise analysis, the mean rCBF from each significant ROI was extracted from all subjects, at both time points.

### Hippocampal analysis

To examine the effect of exercise cessation on hippocampal blood flow, we conducted an *a priori* analysis that was restricted to voxels within the hippocampus. To isolate and examine hippocampal rCBF, we used FreeSurfer's (version 5.3.0) automated subcortical processing stream (Fischl et al., 2002). This procedure segmented the T1-weighted anatomical image and rendered a segmentation map based on both atlas probabilities and subject-specific tissue intensities. The segmentation map was normalized using non-linear transformation to maintain both the accuracy and integrity of the labeled subcortical anatomy. The non-linear transformation parameters were then used to warp the co-registered CBF map to standard space. The normalized bilateral hippocampal regions were extracted, merged together, and used as a mask for the voxel-wise analysis. Using the same clustering procedures as described above, Monte Carlo simulations were run over the hippocampal volume to establish a FWE threshold of *p* < 0.05 with a minimum cluster size of ≥200 mm^3^.

## Results

### The master athlete profile

The master athletes who volunteered for this study are a unique population and should not be considered equivalent to older adults who engage in regular moderate to vigorous intensity leisure-time physical activity. Our participants had a mean continuous endurance training history of ~29 years, and on average were running 59 km per week and training 5 days per week just prior to the baseline testing. They also regularly participated in regional and national endurance competition. Moreover, as a group these master athletes had a $\overset{\cdot }{\text{V}}$O_2max_ above the 90th percentile for their age and sex.

### Gray matter rCBF

**Figure 1:**
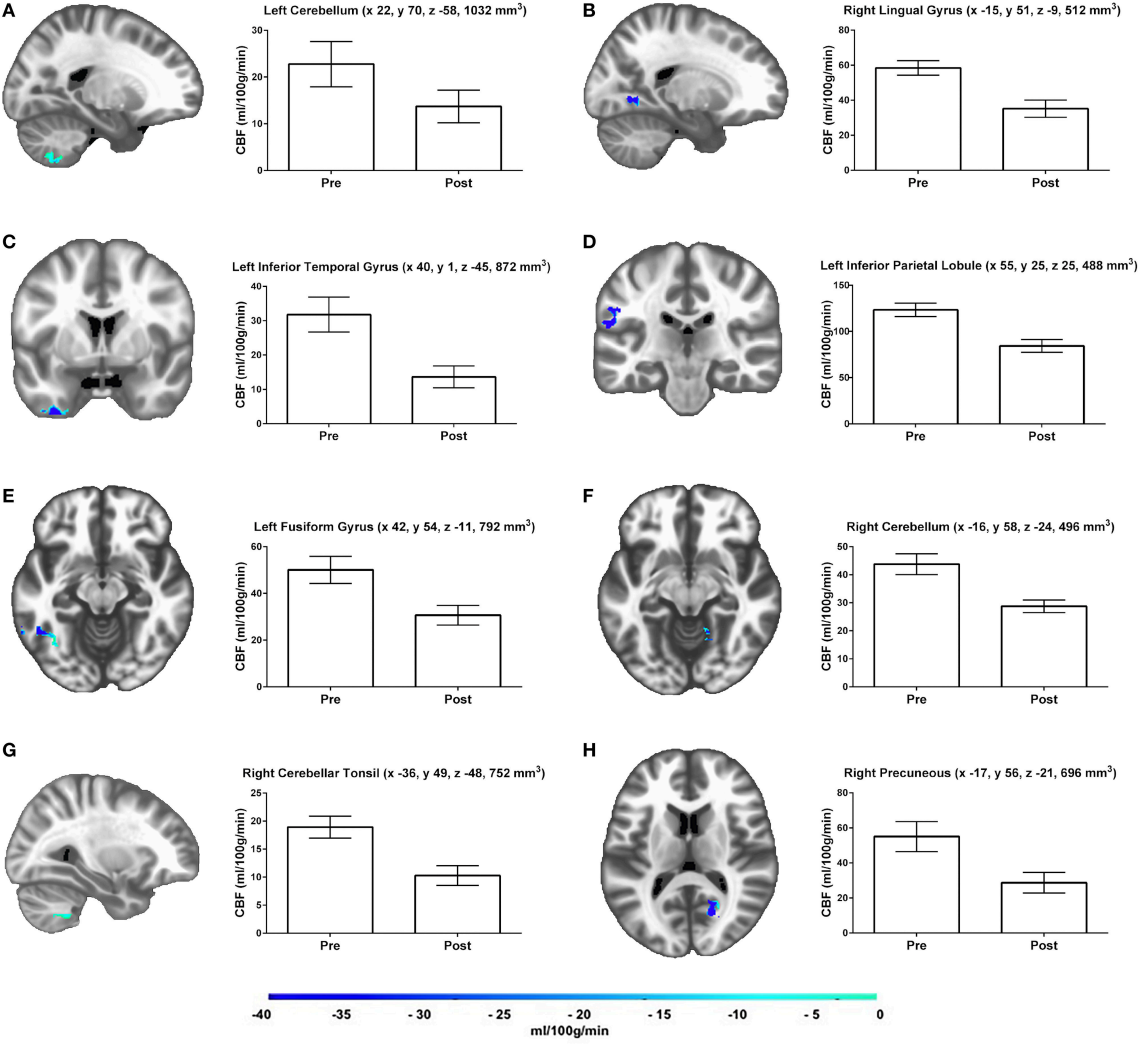
**Results of the gray matter voxel-wise analyses reveal eight brain regions (A–H), which demonstrated significant rCBF changes over time**. Adjacent bar graphs represent the mean rCBF difference within each region, and include brain area, LPI coordinates, and cluster volume in mm^3^. The color bar represents the mean absolute CBF difference (post-detraining minus pre-detraining) within each region, expressed in ml/100g/min. Corrected *p*-values reflect the contrast between the Pre and Post time points.

Results of the gray matter voxel-wise analysis demonstrated that the 10-day exercise cessation period significantly reduced absolute rCBF in eight brain regions (Figure 1). Of note are the Pre > Post comparisons, shown in blue on the Δ rCBF maps in Figure 1, revealing significantly decreased rCBF in each ROI (total volume = 5,640 mm^3^) that remained after correction for multiple comparisons using the False Discovery Rate (see Table 2). These regions included: (L) inferior temporal gyrus, fusiform gyrus, inferior parietal lobule, (R) cerebellar tonsil, lingual gyrus, precuneus, and (L/R) cerebellum. No statistically significant change in whole brain absolute CBF in gray matter was detected [mean (±SD) baseline = 69.4 (±10.4 ml/100g/min), post-cessation = 67.2 (±12.6 ml/100g/min)].

**Table 2:** **Cerebral blood flow (CBF) results from gray matter voxel-wise analysis**.

**#**	**Side**	**Region**	**BA**	***x***	***y***	***z***	**vol**	**Pre CBF (ml/100g/min)**	**Post CBF (ml/100g/min)**	***p***	***η^2^***
A	L	Cerebellum		22	70	−58	1032	22.8 (14.6)	13.7 (10.5)	0.00046	0.801
B	R	Lingual Gyrus	18.19	−15	51	−9	512	58.4 (12.4)	35.2 (14.7)	0.00023	0.833
C	L	Inferior Temporal Gyrus	20	40	1	−45	872	31.8 (15.2)	13.6 (9.6)	0.00058	0.791
D	L	Inferior Parietal Lobule	40	55	25	25	488	123.3 (21.5)	84.3 (20.7)	0.00108	0.756
E	L	Fusiform Gyrus	16	42	54	−11	792	50.1 (17.3)	30.7 (12.6)	0.00056	0.792
F	R	Cerebellum		−16	58	−24	496	43.8 (11.2)	28.7 (6.7)	0.00015	0.849
G	R	Cerebellar Tonsil		−36	49	−48	752	18.9 (5.9)	10.3 (5.3)	0.00008	0.872
H	R	Precuneus	7	−17	56	−21	696	55.0 (25.8)	28.7 (17.5)	0.00036	0.814

*P-values and effect sizes reflect the change from baseline (pre) to after detraining (post) based on the average CBF within each ROI at each time point. η^2^, partial eta squared (effect size); # corresponds with regions shown in Figure 1; BA, Broadmann areas; LPI coordinates, positive, left (x), posterior (y), inferior (z); vol, volume in mm^3^; CBF, cerebral blood flow (ml/100g/min); p, family wise error (FWE) corrected p < 0.05 significance level*.

### Hippocampal rCBF

**Figure 2:**
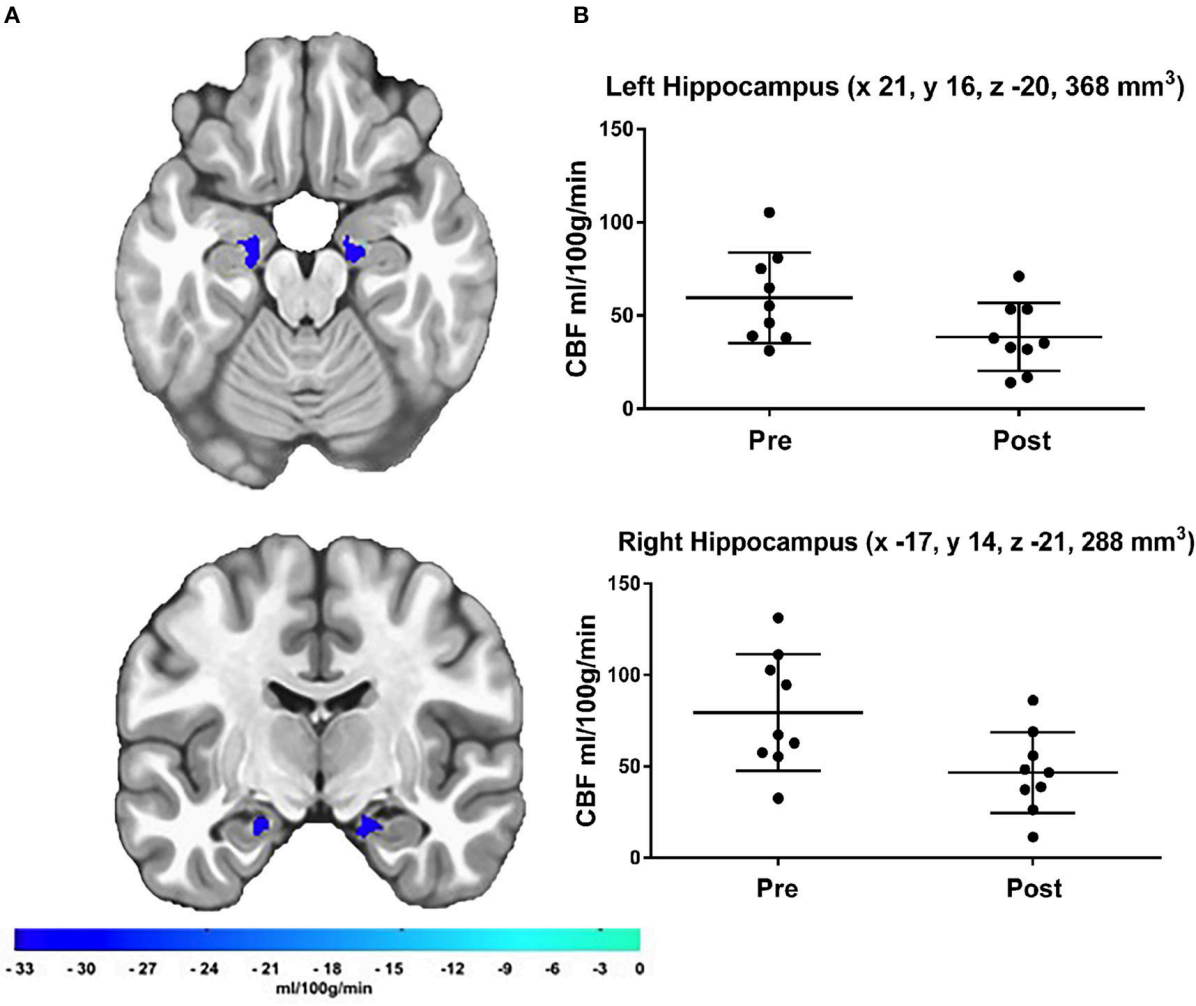
**(A)** Results of the a priori hippocampal analysis demonstrating significant CBF changes over time in the left and right hippocampus. The color bar represents the mean absolute CBF difference (post-detraining minus pre-detraining) within each ROI, **(B)** Scatter plots showing hippocampal CBF for each participant at both time points. Additionally, scatter plots indicate LPI coordinates and cluster volume in mm^3^. Corrected *p*-values were 0.0011 and 0.0041 for left and right hippocampus, respectively.

The *a priori* hippocampal analysis also revealed significantly decreased blood flow in both the left and right hippocampus from before to after the cessation of exercise training (see Figure 2).

### Verbal fluency

Verbal fluency performance did not significantly change from before to after the cessation of training period [mean (±SD) baseline = 19.9 (±4.9 words), post-cessation = 17.4 (±5.8 words), *t*_(8)_ = 0.91, *p* = 0.39].

## Discussion

In the present study, we examined the relationship between short-term exercise cessation and resting rCBF in master athletes. Exercise cessation was associated with reduced rCBF within eight gray matter regions, including bilateral regions of the hippocampus. Importantly, these significant changes were regionally specific, and not the result of global CBF changes after the 10-day period of exercise cessation.

In one of the first studies of its kind, Saltin et al. (1968) demonstrated the deleterious effects of bed rest immobilization on the cardiovascular system. Comparable to decades of biological aging, this extreme sedentary behavior reduced $\overset{\cdot }{\text{V}}$O_2max_ ~27% in just 20 days (Mcguire et al., 2001). Using biopsy samples from the vastus lateralis, Klausen examined the effects of exercise cessation on skeletal muscle. After an 8-week detraining period, both muscle fiber capillarization and oxidative enzyme activity declined (Klausen et al., 1981). Finally, in a study of master athletes, Rogers examined the effects of a short-term period without exercise on insulin-regulated glucose metabolism. After 10 days, ~29% of these older adults developed signs of impaired glucose tolerance or insulin resistance (Rogers et al., 1990). Here, we have extended this literature to demonstrate that not only does exercise cessation among physically fit older adults affect markers of peripheral metabolic function, but also appears to affect brain cortical and hippocampal blood flow.

Exercise training has been shown to robustly affect the structural and functional integrity of the hippocampus in animals and humans, producing neurotrophic effects leading to neurogenesis and angiogenesis in rodents (van Praag et al., 1999; Pereira et al., 2007; Intlekofer and Cotman, 2013), and to increased structural volume in healthy older adults (Erickson et al., 2009). Twelve weeks of exercise training in healthy younger adults was shown to increase the blood volume within the dentate gyrus of the hippocampus and to improve episodic memory performance. Our findings of reduced rCBF bilaterally in the hippocampus suggest that training-induced changes in hippocampal blood flow may be reversed with 10 days of exercise cessation. Our participants, however, did not show any changes in cognitive function over the 10-day cessation of exercise period. Nevertheless, it is not known if cellular adaptations, undetectable using MRI, occur when exercise training is stopped temporarily.

Although several studies have used perfusion-weighted imaging to probe for exercise-induced alterations in rCBF, differences in exercise intensity, study design, and data analysis methods have likely led to conflicting results. In a randomized controlled trial, Chapman et al. (2013) demonstrated that a 12-week aerobic exercise intervention significantly increased rCBF in the anterior cingulate cortex of previously sedentary older adults (Chapman et al., 2013). Likewise, a cross-sectional study by Thomas showed that master athletes had significantly greater rCBF in the posterior cingulate cortex than age-matched sedentary controls (Thomas et al., 2013). While these findings seem to corroborate one another, the effects of acute exercise on rCBF have proven to be complex. Although Smith et al. found rCBF in the motor cortex to be significantly greater after a session of moderate-intensity exercise (Smith et al., 2010), MacIntosh demonstrated, in a similar study, that acute exercise decreased rCBF in the hippocampus and insula (MacIntosh et al., 2014).

Until now, the effects of exercise cessation on cerebrovascular function have been virtually unexplored. This study has extended the literature by showing that a short-term period of physical inactivity among master athletes reduces rCBF in the hippocampus and several gray matter regions. While these effects may have implications for brain function in older adults, it is also possible that these effects represent changes in arterial transit time (Buxton, 2005) or cerebral blood volume (Yoshiura et al., 2009), neither of which we were able to measure. A decrease in arterial transit time, for instance, while using the same post-label delay parameter, could result in an apparent decrease in rCBF (Buxton, 2005). In addition, total blood volume has been shown to increase in response to exercise training (Convertino, 1991) and to decrease after detraining (Coyle et al., 1986). These are important points to consider; however, the regional specificity of the effects we observed suggests these changes were not an artifact of a global flux in blood flow or volume. Although increases in blood volume have been documented in the dentate gyrus after 12 weeks of exercise in younger adults (Pereira et al., 2007), the impact of exercise cessation on cerebral blood volume and arterial transit time has not been established.

Animal research suggests the most viable mechanism for exercise-induced perfusion alterations may be structural modifications to the cerebrovasculature (Swain et al., 2003; Pereira et al., 2007). One such study established these effects by examining the association between increased cerebral blood volume and post-mortem neurogenesis in exercised mice (Pereira et al., 2007). The results indicate that local increases in hippocampal blood volume coincide with the cellular proliferation and reconfiguration within the dentate gyrus. In another investigation, Ryhu provided further support for this proposition by evaluating the effects of both exercise training and exercise cessation on the non-human primate cerebrovascular system (Rhyu et al., 2010). During this study, animals were randomized to either treadmill training or sedentary behavior. After 5 months, a sub-set of primates from the exercise group were subjected to an additional 3 months without exercise. At the 5-month time point, the exercised animals had significantly greater cerebrovascular volumes than their sedentary counterparts. As for the effects of exercise cessation, the increases in vascular volume induced during the exercise-training period were reversed after 3 months of detraining. It is plausible that the cessation of exercise involves a reversal of these effects, which would need to be confirmed in additional animal models.

There are several limitations to the current study. We had a small (*n* = 9) and homogeneous (7 men, ~89% Caucasian) study sample, in which all participants exhibited a high level of aerobic fitness, long history of competitive endurance training, and normal BMI, which limits the generalizability of our findings. While our sample was small, the effects we observed were substantial and all in the same direction. We may have lacked statistical power to detect smaller effects and may have underestimated the decreases in CBF that occurred. In addition, because we only measured rCBF at two time points, we do not know the time course of the changes we observed. It is possible reduced rCBF may have been evident prior to day 10. Further, we cannot speculate regarding whether or not these effects would have increased, decreased or remained stable with a longer period of exercise cessation. We made *a priori* predictions of changes in hippocampal blood flow based on an extensive literature, a method also used by others (Pereira et al., 2007), which avoided adjustment for whole brain voxel-wise comparisons and prevented a possible Type-II error. Nevertheless, future studies should confirm these effects in a larger sample. Additionally, aerobic fitness was assessed only at baseline, so we do not know the magnitude of the fitness change over time. Several studies have documented that in trained individuals substantial decreases in fitness occur after short-term cessation from exercise training (Klausen et al., 1981; Rogers et al., 1990; Mujika and Padilla, 2000b). Finally, the nature of our imaging sequence (single-delay) limited our ability to estimate changes in cerebral blood volume, and possible changes in arterial transit time. Our post label delay values began at 1000 ms and increased at each slice, which does not precisely correspond to the ISMRM Perfusion Study Group recommended guidelines for ASL scanning among older adults (Alsop et al., 2014). This could contribute to CBF quantification challenges, including arterial transit time artifacts. Measuring cerebral blood volume and arterial transit time with multi-delay ASL is an important next step (Alsop et al., 2014).

Approximately two-thirds of the ROIs that showed altered rCBF after exercise cessation are considered part of the brain's default mode network (DMN), which is known to be disrupted with age-related cognitive decline and Alzheimer's disease (Raichle et al., 2001; Sheline et al., 2010; Petrella et al., 2011). Exercise training in older adults has been shown to augment functional connectivity within the DMN in healthy older adults (Voss et al., 2010). While our findings suggest that short-term cessation from endurance training in highly trained older adults may lead to decreased rCBF within the DMN, there was no indication that these effects were detrimental to cognitive function, as measured by a semantic verbal fluency task (which activates brain regions that overlap with the DMN; Binder et al., 2009), or to the integrity of these neural networks. Future exercise detraining studies should examine participants after resuming their training schedules to document whether or not these effects would be reversed. It is also not known if the decreased rCBF we observed solely reflects changes in the rate of blood flow within parenchymal regions, or additionally reflects changes in arterial transit time and/or blood volume. Nevertheless, the exercise cessation paradigm may provide useful information to probe the durability of the effects of exercise training and physical activity on brain function. Just as the effects of long-term endurance exercise training on cardiovascular and metabolic function wane considerably after a short period of detraining (Mujika and Padilla, 2000b), so also may hippocampal and gray matter rCBF be sensitive to exercise cessation.

## Author contributions

Substantial contributions to the conception or design of the work (JH, JS). The acquisition, analysis, or interpretation of data for the work (AA, BL, LW, TS, JS, JH). Drafting the work or revising it critically for important intellectual content (AA, JS, BL, LW, TS, JH).

## Funding

This study was supported by a grant from the National Institutes of Health (HL098810). Its contents are solely the responsibility of the authors and do not necessarily represent the official views of the NIH.

### Conflict of interest statement

The authors declare that the research was conducted in the absence of any commercial or financial relationships that could be construed as a potential conflict of interest.
